# Prevalence of Multimorbidity among Asian Indian, Chinese, and Non-Hispanic White Adults in the United States

**DOI:** 10.3390/ijerph17093336

**Published:** 2020-05-11

**Authors:** Yifan Zhang, Ranjita Misra, Usha Sambamoorthi

**Affiliations:** 1 Department of Pharmaceutical Systems and Policy, West Virginia University School of Pharmacy, Morgantown, WV 26506, USA; usambamoorthi@hsc.wvu.edu; 2 Department of Social & Behavioral Sciences, West Virginia University School of Public Health, Morgantown, WV 26506, USA; ramisra@hsc.wvu.edu; 3 Public Health Training Center, West Virginia University School of Public Health, Morgantown, WV 26506, USA

**Keywords:** multimorbidity, multiple chronic conditions, population-based, National Health Interview Survey, racial disparity, Asian Indians, Chinese, non-Hispanic white

## Abstract

Asian Americans are the fastest-growing minority group in the United States, yet little is known about their multimorbidity. This study examined the association of Asian Indians, Chinese and non-Hispanic whites (NHWs) to multimorbidity, defined as the concurrent presence of two or more chronic conditions in the same individual. We used a cross-sectional design with data from the National Health Interview Survey (2012–2017) of Asian Indians, Chinese, and NHWs (*N* = 132,666). Logistic regressions were used to examine the adjusted association of race/ethnicity to multimorbidity. There were 1.9% Asian Indians, 1.8% Chinese, and 96.3% NHWs. In unadjusted analyses (*p* < 0.001), 17.1% Asian Indians, 17.9% Chinese, and 39.0% NHWs had multimorbidity. Among the dyads, high cholesterol and hypertension were the most common combination of chronic conditions among Asian Indians (32.4%), Chinese (41.0%), and NHWs (20.6%). Asian Indians (AOR = 0.73, 95% CI = (0.61, 0.89)) and Chinese (AOR = 0.63, 95% CI = (0.53, 0.75)) were less likely to have multimorbidity compared to NHWs, after controlling for age, sex, and other risk factors. However, Asian Indians and Chinese were more likely to have high cholesterol and hypertension, risk factors for diabetes and heart disease.

## 1. Introduction

The co-occurrence of multiple health conditions in the same individual, also known as multimorbidity, has become a priority for global health [1]. Original articles, systematic reviews, and meta-analyses [2] have documented the prevalence of multimorbidity in both young and older adults and reported substantial adverse clinical, humanistic, and economic burden. The global burden of multimorbidity is well-established [3]. Multimorbidity is significantly associated with lower worker productivity [4], higher risk of polypharmacy [5,6], impaired functioning [7], frailty [8], poor quality of life [9], substantial higher healthcare use [10,11,12], increased healthcare costs [13,14], and increased risk of death [15]. A systematic review has concluded that multimorbidity is highly prevalent [16]. In addition, the prevalence of multimorbidity is growing [17]. Epidemiologic data suggest differences in multimorbidity prevalence rates by age, sex, race, and socioeconomic status [18,19,20,21,22]. Older adults, racial minorities, and those with low socioeconomic status are at high risk for multimorbidity [23,24,25].

Notably, a handful of studies have reported differences in multimorbidity prevalence among racial and Asian ethnic minorities (e.g., Asian Indians and Chinese). A systematic review of multimorbidity and outcomes in South Asians concluded that the prevalence of multimorbidity is poorly understood in South Asian ethnic groups [26]. A few studies in this area have provided mixed results for the association of race/ethnicity to multimorbidity. For example, in Singapore, both Chinese and Asian Indians had higher risks of multimorbidity compared to the native population (Malayans) [27]. In the United States (U.S.), using Hawaii Medicare data, Lim et al. reported that racial minorities (Asians/Pacific Islanders, Hispanics, and Others) had significantly higher prevalence rates of multimorbidity compared to non-Hispanic whites (NHWs) [24]. However, among the residents of Olmsted County, Minnesota, Asians had a lower prevalence of physical multimorbidity (12.7% vs. 26.7%) and physical and mental health multimorbidity (3.2% vs. 8.6%) compared to whites [28,29]. These studies have limitations because Asian Americans that represent many heterogeneous subgroups were combined [30]. In 2016, a data brief reported by Bloom and Black [31] looked at the health status among Asian subgroups. They found that Asian Indian (16.9%) and Chinese adults (11.3%) were less likely than all U.S. adults (24.1%) to have multimorbidity. However, they did not examine risk factors associated with multimorbidity other than age. Thus, questions remain regarding Asian ethnic disparities in multimorbidity prevalence rates.

Specifically for Asian populations living in the U.S., a focus on multimorbidity is overdue and necessary for several reasons: the Asian population has increased substantially during the past twenty years in the United States, from less than 2% of the total population prior to 2000 to nearly 7% as of 2018 [32,33,34]. Among them, Chinese and Asian Indians are the two largest subgroups. This tremendous growth, as well as lack of adequate knowledge of their physical and mental health, presents challenges for healthcare professionals, policymakers, and others. 

Asian Americans are a heterogeneous group with differing levels of education, household income, and language ability. According to the American Community Survey, 74.6% of Asian Indians had a bachelor’s degree or higher, while only 57.2% of the Chinese did the same. The median household income is $116,793 for Asian Indians and $81,487 for Chinese. A smaller amount (17.3%) of Asian Indians speak English less than “very well” compared to 36.9% of Chinese [35]. Researchers and advocacy groups have emphasized the importance of collecting and reporting data by specific Asian subgroups [30,36,37]. Acknowledging this importance, as of 2010, Section 4302 of the Affordable Care Act [38] requires that all health surveys sponsored by the Department of Health and Human Services (DHHS) include standardized information on race/ethnicity at a granular level among the Asian sub-population [39].

However, Asian subgroups are still frequently combined with Pacific Islanders [40,41], or into a single Asian category [29,42], masking heterogeneity among the subgroups [43]. Other studies that have explored the prevalence of multimorbidity in Asians subgroups either had small sample sizes [37] or were restricted to specific geographic areas [28,29], limiting the generalizability of the results. Hence, using a representative sample to understand the patterns of health by specific Asian ethnic subgroups (e.g., Asian Indians and Chinese) is needed.

We did not include other racial/ethnic groups such as Hispanic or Latinos and African Americans because many studies have compared multimorbidity prevalence among Hispanics and NHWs and African Americans and NHWs [44,45,46,47]. These studies reported that Non-Hispanic Blacks had greater odds of multimorbidity compared to NHWs after adjusting for other risk factors. However, Hispanic adults and Non-Hispanics of other races were less likely to have multimorbidity when compared to NHWs. To avoid repeated work, we did not include Hispanic Americans or African Americans in our study.

Therefore, the purpose of this study was to estimate the prevalence of multimorbidity among Asian Indians, Chinese, and NHWs and examine adjusted associations of race/ethnicity to multimorbidity after controlling for specific characteristics that are known to be associated with multimorbidity.

## 2. Materials and Methods 

### 2.1. Study Design

We used a cross-sectional design with data on adults from the following racial/ethnic groups: Asian Indians, Chinese, and NHWs.

### 2.2. Data Source

We used data from the National Health Interview Survey (NHIS). NHIS is an annual cross-sectional survey of a nationally representative sample of noninstitutionalized civilian households and members of the households. It has been conducted continuously since 1957 [48]. The NHIS collects information on a comprehensive list of health topics, including chronic conditions, health status, healthcare services, and health behaviors and their distribution by demographic, socioeconomic, and access to care characteristics through personal household interviews [48]. Since 1992, the NHIS has collected disaggregated data on Asians. Furthermore, in the 2006 survey year, the NHIS implemented oversampling of Asian households for the first time [49]. As of 2011, NHIS followed the policy guidelines from the DHHS to standardize the collection of race/ethnicity data at a granular level among the Asian sub-population [39].

In this study, we combined information from the family core, the sample adult core, and the person files using established National Center for Health Statistics guidelines for combining NHIS data [50]. To ensure adequate Asian Indian and Chinese sample sizes, we combined 2012–2017 annual files for pooled analyses of the NHIS. The data is de-identified and open to the public [51]. This study was not considered human subjects research.

### 2.3. Analytical Sample

We restricted the study sample to adults (age ≥ 18 years) and the following racial/ethnic groups: Asian Indians, Chinese, and NHWs. We only included adults who participated in the sample adult core (*N* = 132,694) and excluded adults who had missing data for multimorbidity (*N* = 28). We pooled multiple years of NHIS (2012–2017) to ensure an adequate sample size for the Asian Indian and Chinese subgroups. The final sample size was 132,666. When weighted to the U.S. population, the sample represented 1.9% Asian Indians, 1.8% Chinese, and 96.3% NHWs.

### 2.4. Measures

#### 2.4.1. Dependent Variable: Presence of Multimorbidity

Although there is a consensus that multimorbidity is the co-existence of multiple health conditions in the same individual, there is no uniform definition of multimorbidity [52]. In our study, we defined multimorbidity as the presence of two or more chronic conditions from the following 10 chronic physical conditions: arthritis, asthma, cancer, chronic obstructive pulmonary disease (COPD), chronic kidney disease, diabetes, high cholesterol, hypertension, heart disease, and stroke. We selected these conditions based on the DHHS strategic framework for program, policy, and research [53]. These conditions were derived from affirmative responses to questions that asked a respondent whether he/she has (1) EVER been told by a doctor or other health professional that she/he had the chronic condition, (2) or during the past 12 months, been told by a doctor or other health professional that she/he had the chronic condition.

#### 2.4.2. Key Independent Variable: Race/Ethnicity—Asian Indians, Chinese, and NHWs

Race/Ethnicity was categorized based on an individual’s response to questions that asked the respondent (1) whether he/she was Hispanic, Latino/a, or Spanish origin, and (2) what his/her race was. Individuals responded both “not of Hispanic, Latino/a, or Spanish origin” and “White” were categorized as NHWs. For Asian subgroups, there were four subcategories available in NHIS public-use data files: Asian Indian, Chinese, Filipino, and other Asian. In this study, we included individuals who self-identified as Asian Indians or Chinese.

#### 2.4.3. Other Independent Variables

The selection of other independent variables was guided by adapted determinants of health models [54,55,56,57,58]. The independent variables included: biological characteristics (age and sex), socioeconomic status (education, employment, poverty status measured by the percentage of household income to the federal poverty line (FPL)), and access to care (presence of health insurance and doctor’s office visits in the past 12 months). Age was categorized into four groups: 18–39 years, 40–49 years, 50–64 years, and ≥65 years. Education was categorized as less than high school, high school (or equivalent), some college, and college (bachelor’s degree or higher). Employment status was categorized as employed (with a job or business) and not employed (not working at a job or business, looking for work, or working at a family owned business with no pay), based on an individual’s response to the question that asked the respondent what he/she was doing last week. 

We also included marital status and depressive symptoms as independent variables because existing literature has shown that those two variables are associated with chronic diseases [59,60]. Marital status was defined as married (married or living with partner), never married, and separated/widowed/divorced. Depressive symptoms were measured based on an individual’s response to a question that asked for the frequency of feeling so sad that nothing cheers her/him up in the past 30 days. Individuals who reported all of the time, most of the time or some of the time, were categorized as having depressive symptoms, while individuals reported a little of the time or none of the time were categorized as having no depressive symptoms.

As many chronic conditions share similar behavioral risk factors (smoking, alcohol use, obesity, and physical inactivity), we also included behavioral factors. Smoking and alcohol use were categorized into three groups: former user, current user, and never used. Physical activity was measured by self-reported frequency of vigorous activity and was categorized as daily, weekly, and physically inactive (monthly/yearly/never/unable). Obesity was measured by Body Mass Index (BMI), which was calculated from self-reported height and weight and adjusted for the Asian population. In NHW, overweight was defined as a BMI between 25 to 30 kg/m^2^ and obese BMI 30 kg/m^2^ or greater. In Asian Indian and Chinese, overweight was defined as a BMI between 23.0 to <25 kg/m^2^, and obesity BMI 25 kg/m^2^ or greater, as recommended by the World Health Organization guidelines [61]. Given the unique Asian American immigration experience, one additional variable was included in the regression analysis: born outside the United States. As historical immigration streams placed Asian Indians and Chinese in specific regions of the U.S. [62], we also adjusted for the region (Northeast, South, Midwest, and West) of the U.S. The interview year was included to control for potential confounding.

### 2.5. Statistical Analysis

As NHIS involves a complex, multistage probability sample that incorporates stratification and clustering, we used survey procedures in the analyses. Sampling weights were constructed by dividing the existing person file weights by 6, as 6 years of data were combined [63,64]. Significant differences in individual characteristics among racial/ethnic groups and multimorbidity were tested with Rao-Scott chi-square tests. Multivariate logistic regressions were used to examine the associations of Asian Indians/Chinese/NHWs to multimorbidity after adjusting for sex, age, education, employment, poverty status, marital status, access to care, obesity, smoking, alcohol use, physical activity, depressive symptoms, region, foreign-born status and NHIS year. Differences in multimorbidity between Asian Indians and Chinese were also assessed using the unadjusted and fully adjusted model.

Results from logistic regressions were reported in terms of unadjusted (UORs) and adjusted odds ratios (AORs) and associated 95% confidence intervals (CIs). As multimorbidity is known to increase with age, the results were stratified by age (≥65 years vs. <65 years). Sixty-five years was selected as the age cutoff because of the availability of national health insurance (i.e., Medicare) for all eligible individuals aged 65 years or older.

## 3. Results

In our sample, 51.5% were female, 22.2% were 65 years or older; 42.9% had high income (FPL > 400%), and 35.6% had college education. The majority of individuals had health insurance (91.2%). Some behavioral risk factors reported by the participants were obese (28.0%), current smokers (17.2%), and physical inactivity (54.2%). These data are not shown in a table.

### 3.1. Description of Characteristics among Asian Indians, Chinese, and NHWs

**Figure 1:**
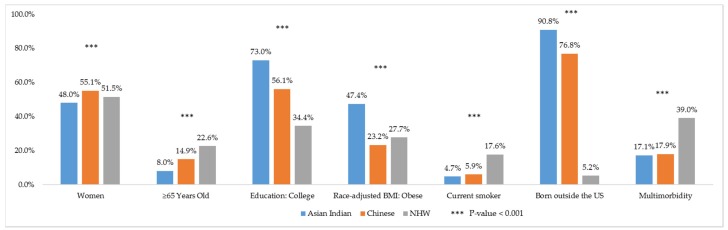
Selected sample characteristics in weighted % among Asian Indian, Chinese, and Non-Hispanic White adults (age ≥ 18 years). National Health Interview Survey, 2012–2017.

Selected characteristics among Asian Indians, Chinese, and NHWs are presented in Figure 1. There were statistically significant differences in all characteristics except health insurance coverage across the three groups (Table 1). In terms of biological characteristics, NHW had the highest percentage (22.6%) of older individuals (age > 65 years) followed by Chinese (14.9%) and Asian Indians (8.0%). For socioeconomic status, Asian Indians had the highest percentage of college education (73.0%), followed by Chinese (56.1%) and NHWs (34.4%). For behavioral risk factors, NHWs had the highest rates of current smoking (17.6%) and alcohol use (69.5%). Asian Indians had the lowest rates of current smoking (4.7%), alcohol use (43.8%), and physical inactivity (50.4%). Yet, Asian Indians had the highest rates of obesity (47.4%). Geographically, higher numbers of Asian Indians resided in the South and Western regions, while the Chinese largely resided in the Northeastern and Western U.S. 

### 3.2. Prevalence of Multimorbidity among Asian Indians, Chinese, and NHWs

Unweighted numbers and weighted percentages of multimorbidity by individual characteristics, including racial/ethnic groups, are presented in Table 2. Overall, 38.2% had multimorbidity. A lower percent of Asian Indians (17.1%) and Chinese (17.9%) had multimorbidity compared to NHWs (39.0%). Unadjusted logistic regressions revealed that Asian Indians (UOR = 0.32, 95% CI = (0.27, 0.38)) and Chinese (UOR = 0.34, 95% CI = (0.30, 0.39)) were less likely to have multimorbidity compared to NHWs (Table 3). 

### 3.3. Chronic Condition Combinations among Asian Indians, Chinese, and NHWs

Among those with two chronic conditions (dyads), high cholesterol & hypertension was the most common combination across Asian Indians (32.4%), Chinese (41.0%) and NHWs (20.6%). Arthritis & hypertension was the second most common combination among Asian Indians (13.6%), Chinese (11.0%), and NHWs (13.6%). The third most common combination differed across the racial/ethnic groups with diabetes & high cholesterol in Asian Indians (10.1%), asthma & high cholesterol in Chinese (8.6%) and arthritis & high cholesterol in NHWs (10.9%).

Among three chronic conditions (triads), both Asian Indians and Chinese shared the same top three combinations: diabetes & high cholesterol & hypertension (30.4%, 21.9%), arthritis & high cholesterol & hypertension (16.7%, 20.0%), and heart disease & high cholesterol & hypertension (10.3%, 10.1%). In NHWs, arthritis & high cholesterol & hypertension was the most common combination (18.9%), followed by heart disease & high cholesterol & hypertension (8.0%). Diabetes & high cholesterol & hypertension (6.9%), which was the most common triad among Asian Indians and Chinese, ranked third in NHWs.

### 3.4. Adjusted Associations of Race/Ethnicity to Multimorbidity

When adjusted for age and sex, Asian Indians (AOR = 0.50, 95% CI = (0.42, 0.59)) and Chinese (AOR = 0.36, 95% CI = (0.32, 0.42) continued to show lower likelihood of having multimorbidity than NHWs (Table 3). In the fully adjusted regression model, that controlled for age, sex, marital status, education, employment, poverty status, access to care, race-adjusted BMI, physical activity, smoking, alcohol use, depressive symptoms, region, foreign-born status and NHIS year, Asian Indians (AOR = 0.73, 95% CI = (0.61, 0.89)) and Chinese (AOR = 0.63, 95% CI = (0.53, 0.75)) were less likely to have multimorbidity than NHWs. It has to be noted that in these regressions, missing indicators were included for variables with missing data (education, poverty status, marital status, employment, health insurance, doctor’s office visit, race-adjusted BMI, physical activity, smoking, alcohol use, depressive symptoms, and foreign-born status).

We observed that women (AOR = 0.81, 95% CI = (0.78, 0.85)) and individuals who were born outside the US (AOR = 0.70, 95% CI = (0.64, 0.76)) were less likely to have multimorbidity compared to men and individuals who were born in the U.S. Older adults (all subgroups aged 40 years or older), those who were not employed (AOR = 1.50, 95% CI = (1.43, 1.56)), without college education (all subgroups), with household income <100% FPL (AOR = 1.38, 95% CI = (1.28, 1.49)) or 100–200% FPL (AOR = 1.21, 95% CI = (1.13, 1.30)), with overweight (AOR = 1.70, 95% CI = (1.62, 1.78)) or obesity (AOR = 3.07, 95% CI = (2.92, 3.23)), those who reported physical inactivity (AOR = 1.25, 95% = (1.15, 1.35)), current smokers (AOR = 1.44, 95% CI = (1.37, 1.52)), and those who visited the doctor’s office (all subgroups) were more likely to report multimorbidity compared to the reference groups (18–39 years, employed, college education, FPL ≥ 400%, normal/underweight BMI, daily physical activity, non-smokers, and no visit to the doctor’s office). We also observed that those with self-reported depressive symptoms (all of the time, most of the time or some of the time) were more likely to have multimorbidity (AOR = 1.64, 95% CI = (1.54, 1.74)) compared to those reporting no symptoms (a little of the time or none of the time).

**Figure 2:**
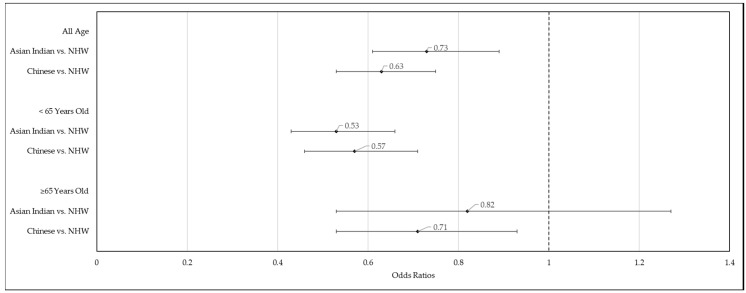
Adjusted Odds Ratios (AOR) and 95% Confidence Intervals (CI) of racial/ethnic categories from logistic regression on multimorbidity among Asian Indian, Chinese, Non-Hispanic White (NHW) adults (age ≥ 18 years) stratified by age group (<65 years vs. ≥65 years). National Health Interview Survey, 2012–2017.

When stratified by age (≥65 years vs. <65 years), Asian Indians and Chinese were less likely to have multimorbidity compared to NHWs (Figure 2). Among the younger age group (<65 years), the AORs for Asian Indians and Chinese were 0.53 (95% CI = (0.43, 0.66)) and 0.57 (95% CI = (0.46, 0.71)) respectively. Among the older age group (≥65 years), the AORs for Asian Indians and Chinese were 0.82 (95% CI = (0.53, 1.27)) and 0.71 (95% CI = (0.53, 0.93)), respectively. The odds ratio for Asian Indians was no longer significant (*p*-value = 0.370) among the older age group.

### 3.5. Asian Indians and Chinese—Comparison of Multimorbidity

Our study found similar prevalence rates of multimorbidity among Asian Indians (17.1%) and Chinese (17.9%). Compared to Chinese, Asian Indians had a lower percentage of current smokers, alcohol use, and being physically inactive. Yet, Asian Indians were found to have a higher prevalence of high cholesterol and diabetes. Among those with two conditions, 41.0% of the Chinese had a combination of high cholesterol and hypertension, compared to 32.4% of Asian Indians. The unadjusted model and the fully adjusted model showed no significant differences in multimorbidity between Asian Indians and Chinese (UOR = 0.94, 95% CI = (0.77, 1.16); AOR = 1.17, 95% CI = (0.92, 1.48); reference = Chinese). This result is not shown in a table.

## 4. Discussion

This is the first study to thoroughly investigate multimorbidity in Asian Indians and Chinese in the US, the two largest Asian subgroups, using a national representative data. Our study findings provide new information that even after controlling for all the relevant factors, including foreign-born status, multimorbidity among Asian Indians and Chinese were lower compared to NHWs. Existing studies have used a single disease framework and documented a high prevalence of diabetes among Asian Americans [65], specifically among Asian Indians [66]. Our study extended published literature by examining multimorbidity as well as combinations of conditions. Our study findings suggest that public health programs, research, and practice need to consider epidemiologic characteristics, including race/ethnicity, to reduce the risk of multimorbidity and in its management.

Our findings are consistent with the lower prevalence rates of multimorbidity observed in other published studies that have combined all Asians into one group. For example, Machlin and Soni [67] estimated that Asians had 16.2% of treated prevalence for multimorbidity compared to 28.5% in NHWs, using the 2009 Medical Expenditure Panel Survey data. St Sauver et al. [42] reported that the standardized incidence of multimorbidity was lower in Asians (men 29.5%, women 34.9%) compared to Whites (men 36.0%, women 39.4%).

One could attribute favorable socioeconomic status and the healthy immigrant effect [68] to low rates of multimorbidity among Asian Indians and Chinese. In our study, Asian Indians and Chinese had higher levels of education and income compared to NHWs. A systematic review of 24 studies suggests that high levels of education and income are associated with a lower likelihood of multimorbidity [69]. However, this may be less of a factor among older adults. In our study, among older adults (age ≥ 65 years), the AORs of multimorbidity for Asian Indians and Chinese were 0.82 and 0.71, respectively, compared to the AORs among young Asian Indians and Chinese (0.53 and 0.57). This may be due to the convergence in natives’ health vs. immigrants’ health with years since immigration due to acculturation [70].

Furthermore, individuals from Asian cultures also tend to have different healthcare use rates. It is not a cultural norm to receive routine checkups and regular preventive care [71]. Additionally, language is a major barrier for Asian Americans seeking care, especially for many elders and recent migrants [71]. The language barrier and cultural attitudes about healthcare may lead to underreporting of their chronic condition until the problem becomes imminent. This could result in an underestimation of multimorbidity prevalence in Asian Indian and Chinese. Furthermore, Asian Indian and Chinese may have a healthier dietary habit and lower fast-food intake [72,73] that have contributed to their lower multimorbidity rates.

In our study, 17.1% of Asian Indians and 17.4% of Chinses had multimorbidity. Yet, the reported lower rates among Asian Indians and Chinese might not reflect the holistic picture of health status in these two groups. A study using the 2009 Nationwide Inpatient Sample (NIS) found that Asian/Pacific Islanders had the highest mortality compared to other races/ethnicities, regardless of the number of chronic conditions [41]. As there is well-established literature that multimorbidity can lead to poor health outcomes including high mortality, hospitalizations, poor quality of life, and increased costs, future research needs to explore the effect of multimorbidity among Asian Indians and Chinese on these prior health outcomes, as well as mediating and moderating factors of such negative outcomes. 

Furthermore, we did not find any significant differences in multimorbidity between Asian Indians and Chinese. The two groups also share some specific combinations of chronic conditions (high cholesterol & hypertension, and high cholesterol & hypertension & diabetes), that had a higher prevalence among them compared to NHW. Both high cholesterol and hypertension are risk factors for diabetes and heart disease. While the high prevalence of diabetes and heart disease among Asian Indians is well established [74,75,76], a significantly lower risk for coronary heart disease was found for Chinese compared to NHWs [77]. Moreover, a study examining functional limitations among middle-aged and older adults reported that Asian Indians had higher odds for functional limitations compared to Chinese [78]. Future studies need to explore whether the association of multimorbidity to health outcomes remain similar among the two groups and identify specific factors that may affect the health outcomes of the two groups differentially. Programs and policies targeted towards multimorbidity may need to consider specific combinations of chronic conditions and their outcomes in addition to a global definition of multimorbidity.

Our results showed that 39.0% of NHW adults had multimorbidity, higher than other published studies that used the NHIS data [31,44,45,79]. The discrepancy may be caused by differences in the study period, age composition, and the selection of chronic conditions. For example, the study period of Ward and Schiller 2013 [45] was from 2001 to 2010. Another study, Johnson-Lawrence et al. 2017 [44], was restricted to individuals aged 30–64 years old. Both studies and Bloom and Black 2016 [31] did not include high cholesterol as a chronic condition when defining multimorbidity. Our study included 9 of the 20 proposed DHHS conditions, excluded hepatitis, and included high cholesterol. The expanded selection of chronic conditions may have resulted in a higher percentage of multimorbidity than the rates reported in previous studies.

The results presented here are tempered by several caveats, and they must be considered in the interpretation of our findings. First, multimorbidity was generated by self-reported health conditions in the NHIS. Thus, the prevalence of health conditions may be overestimated or underestimated. This limitation is inherent in the NHIS as all information is obtained through self-report. The multimorbidity measure has further limitations. Although it is a standard approach that has been used in other studies of multimorbidity [45,80,81,82], this dichotomized variable does not account for the severity, complexity, or duration of the chronic conditions studied. Second, independent variables were limited to the variables available in NHIS. We included factors that were known to be risk factors of multimorbidity. Other factors, such as diet, which may also affect the risk of multimorbidity, were not included in the multivariate model. Third, the cross-sectional nature of our study precludes us from making any causal inferences. Without the information on temporal relationship, we could not infer causality on the observed association between risk factors and the prevalence of multimorbidity. Nonetheless, our study contributed to the nascent literature on multimorbidity among Asian subgroups by using a nationally represented Asian Indians, Chinese, and NHWs in the US. In our analyses, we controlled for a host of covariates not considered in previous studies of race/ethnicity with multimorbidity, including doctor’s office visit, region and foreign-born status. In addition, we stratified the results by age group, which allowed us to test for the independent associations of these variables with multimorbidity among elderly and non-elderly groups. Moreover, in 2012–2017 NHIS data, Asian persons were oversampled to allow for better estimation of the health characteristics of these populations [83,84,85,86,87,88]. By pooling six years of NHIS data, we were able to raise the sample size and further increase the precision of our study while ensuring validity (i.e., representativeness) in measuring multimorbidity among the entire population of Asian Indians, Chinese and NHWs in the U.S. 

## 5. Conclusions

We observed that Asian Indians and Chinese had lower prevalence rates of multimorbidity compared to NHWs after accounting for age, sex, socioeconomic characteristics, and health behaviors. Yet, Asian Indians and Chinese were more likely to have specific combinations of high cholesterol and hypertension, risk factors for heart disease and diabetes. Future studies on types of multimorbidity and their associated health outcomes, especially those related to cardiovascular clusters among Asian subgroups, are warranted.

## Figures and Tables

**Table 1:** Unweighted *N* and weighted % of characteristics among Asian Indians, Chinese and Non-Hispanic White (NHW) adults (age ≥ 18 years). National Health Interview Survey, 2012–2017.

ALL		Asian Indian		Chinese		NHW		*p*-Value
		*N*	Wt%	*N*	Wt%	*N*	Wt%	
		2297	100.0	2403	100.0	127,966	100.0	
**Multimorbidity**								<0.001
	No (0–1 chronic conditions)	1946	82.9	1941	82.1	73,368	61.0	
	Yes (2–3 chronic conditions)	278	14.1	372	14.9	36,360	26.8	
	Yes (4+ chronic conditions)	73	3.0	90	3.0	18,238	12.2	
**Age in Years**								<0.001
	18–39	1376	53.5	1107	42.1	37,960	33.5	
	40–49	421	21.0	429	21.7	18,489	16.1	
	50–64	317	17.5	448	21.3	35,426	27.8	
	≥65	183	8.0	419	14.9	36,091	22.6	
**Poverty Status**								<0.001
	<100% FPL	253	8.2	484	15.5	13,801	8.4	
	100%–<200% FPL	244	10.0	311	12.3	20,102	13.7	
	200%–<400% FPL	419	19.6	458	18.8	34,928	26.9	
	≥400% FPL	1193	54.3	917	42.9	48,655	42.7	
**Employment**								<0.001
	Employed	1599	68.5	1381	60.7	72,428	60.0	
	Not employed	696	31.4	1021	39.3	55,476	40.0	
**Health Insurance**								0.177
	Insured	2102	91.6	2180	91.7	116,624	91.2	
	Not insured	188	8.1	204	7.6	10,989	8.5	
**Marital Status**								<0.001
	Married	1564	77.3	1263	64.1	67,217	63.5	
	Separated/widowed/divorced	166	5.5	322	9.0	35,667	17.9	
	Never married	564	17.2	812	26.7	24,818	18.5	
**Doctor’s Office Visit**								<0.001
	No visit	530	21.1	566	21.2	18,307	14.7	
	1 visit	576	26.3	533	24.7	21,234	17.1	
	2–3 visits	620	28.3	631	26.9	33,995	26.9	
	4 and more visits	528	22.4	631	25.7	52,422	39.7	
**Alcohol Use**								<0.001
	Abstained	1094	49.4	990	41.1	18,675	14.8	
	Former drinker	112	5.3	171	6.9	20,378	14.3	
	Current drinker	1061	43.8	1218	51.0	87,159	69.5	
**Physical Activity**								<0.001
	Daily	160	6.9	112	4.8	8779	7.0	
	Weekly	983	41.3	925	36.4	45,769	37.6	
	Monthly/yearly/never/unable	1129	50.4	1341	57.9	71,953	54.2	
**Depressive Symptoms**								0.018
	All/most/some of the time	209	8.9	219	9.2	13,477	9.8	
	A little/none of the time	2006	86.9	2108	88.0	111,034	87.4	
**Region**								<0.001
	Northeast	512	24.7	587	28.5	23,151	19.0	
	Midwest	402	17.1	265	10.0	33,989	27.5	
	South	753	32.0	374	15.0	40,671	33.7	
	West	630	26.2	1177	46.5	30,155	19.7	
**NHIS Year**								0.003
	2012	404	13.3	449	14.1	20,838	16.6	
	2013	415	14.6	458	16.2	20,795	16.6	
	2014	400	16.0	456	16.5	23,052	16.7	
	2015	417	17.6	420	16.8	21,072	16.7	
	2016	351	20.0	329	17.3	23,370	16.7	
	2017	310	18.5	291	19.1	18,839	16.7	

Based on 132,666 adult (age ≥ 18 years) NHIS participants from pooled cross-sectional data for years from 2012 through 2017, belonging to the racial/ethnic groups (Asian Indian, Chinese, and NHW) and did not have missing data on multimorbidity. Statistically significant differences by racial/ethnic groups were tested with Rao-Scott chi-square tests. Numbers may not add to total due to missing data in education, poverty status, employment, health insurance, marital status, doctor’s office visit, race-adjusted BMI, smoking, alcohol use, physical activity, and depressive symptoms.

**Table 2:** Unweighted *N* and weighted % of Multimorbidity among Asian Indian, Chinese, Non-Hispanic White (NHW) adults (age ≥ 18 years). National Health Interview Survey, 2012–2017.

ALL		Multimorbidity		No Multimorbidity		*p*-Value
		*N*	Wt%	*N*	Wt%	
		55,411	100%	77,255	100%	
**Sex**						0.001
	Women	30,831	38.8	40,838	61.2	
	Men	24,580	37.7	36,417	62.3	
**Age in Years**						<0.001
	18–39	4352	10.4	36,091	89.6	
	40–49	5384	27.5	13,955	72.5	
	50–64	18,381	49.9	17,810	50.1	
	≥65	27,294	74.3	9399	25.7	
**Race/Ethnicity**						<0.001
	Asian Indian	351	17.1	1946	82.9	
	Chinese	462	17.9	1941	82.1	
	NHW	54,598	39.0	73,368	61.0	
**Education**						<0.001
	Less than high school	6676	51.1	4847	48.9	
	High school	15,593	43.6	16,941	56.4	
	Some college	17,430	38.1	24,749	61.9	
	College	15,551	31.6	30,505	68.4	
**Poverty Status**						<0.001
	<100% FPL	5983	38.7	8555	61.3	
	100%–<200% FPL	10,088	44.1	10,569	55.9	
	200%–<400% FPL	15,217	38.8	20,588	61.2	
	≥400% FPL	19,121	35.2	31,644	64.8	
**Employment**						<0.001
	Employed	20,666	26.2	54,742	73.8	
	Not employed	34,730	56.4	22,463	43.6	
**Health Insurance**						<0.001
	Insured	52,624	39.7	68,282	60.3	
	Not insured	2712	22.8	8669	77.2	
**Marital Status**						<0.001
	Married	28,128	39.0	41,916	61.0	
	Separated/widowed/divorced	21,655	58.3	14,500	41.7	
	Never married	5528	16.8	20,666	83.2	
**Doctor’s Office Visit**						<0.001
	No visit	2740	13.0	16,663	87.0	
	1 visit	5396	21.1	16,947	78.9	
	2–3 visits	14,113	36.3	21,133	63.7	
	4 and more visits	32,253	56.8	21,328	43.2	
**Race-adjusted BMI**						< 0.001
	Underweight/normal	14,229	26.1	33,139	73.9	
	Overweight	18,726	39.8	24,772	60.2	
	Obese	20,416	51.6	16,762	48.4	
**Smoking**						<0.001
	Never smoked	25,912	31.8	47,308	68.2	
	Former smoker	19,737	53.1	15,612	46.9	
	Current smoker	9496	37.6	13,974	62.4	
**Alcohol Use**						<0.001
	Abstained	8968	36.8	11,791	63.2	
	Former drinker	12,561	57.9	8100	42.1	
	Current drinker	33,185	34.6	56,253	65.4	
**Physical Activity**						<0.001
	Daily	2906	29.2	6145	70.8	
	Weekly	13,230	26.2	34,447	73.8	
	Monthly/yearly/never/unable	38,700	47.8	35,723	52.2	
**Depressive Symptoms**						<0.001
	All/most/some of the time	8025	54.2	5880	45.8	
	A little/none of the time	45,895	36.5	69,253	63.5	
**Region**						<0.001
	Northeast	10,385	38.0	13,865	62.0	
	Midwest	14,416	38.0	20,240	62.0	
	South	18,155	40.1	23,643	59.9	
	West	12,455	35.6	19,507	64.4	
**Foreign-Born Status**						<0.001
	Born in the U.S.	52,654	39.3	70,298	60.7	
	Born outside the U.S.	2741	26.5	6899	73.5	
**NHIS Year**						<0.001
	2012	8716	37.7	12,975	62.3	
	2013	8377	36.0	13,291	64.0	
	2014	10,004	38.5	13,904	61.5	
	2015	9257	38.5	12,652	61.5	
	2016	10,478	39.3	13,572	60.7	
	2017	8579	39.4	10,861	60.6	

Based on 132,666 adult (age ≥ 18 years) NHIS participants from pooled cross-sectional data for years from 2012 through 2017, belonging to the racial/ethnic groups (Asian Indian, Chinese, and NHW) and did not have missing data on multimorbidity. Statistically significant differences by multimorbidity were tested with Rao-Scott chi-square tests. Numbers may not add to total due to missing data in education, poverty status, employment, health insurance, marital status, doctor’s office visit, race-adjusted BMI, smoking, alcohol use, physical activity, depressive symptoms, and foreign-born status.

**Table 3:** Unadjusted Odds Ratios (UOR) and Adjusted Odds Ratios (AOR) and 95% Confidence Intervals (CI) of racial/ethnic categories from logistic regression on multimorbidity among Asian Indian, Chinese, and Non-Hispanic White (NHW) adults (age ≥ 18 years). National Health Interview Survey, 2012–2017.

Logistic Regression Model		UOR	95% CI	*p*-Value
**Model 1—Unadjusted**				
**Racial/Ethnic Categories**				
	Asian Indian	0.32	(0.27, 0.38)	<0.001
	Chinese	0.34	(0.30, 0.39)	<0.001
	*NHW (Ref)*			
		**AOR**	**95% CI**	***p*-value**
**Model 2—adjusted for sex and age**				
**Racial/Ethnic Categories**				
	Asian Indian	0.50	(0.42, 0.59)	<0.001
	Chinese	0.36	(0.32, 0.42)	<0.001
	*NHW (Ref)*			
**Model 3—adjusted for sex, age, education, poverty status, employment status, marital status, health insurance, doctor’s office visit, race-adjusted BMI, physical activity, smoking and alcohol use, depressive symptoms, region, foreign-born status and NHIS year**				
**Racial/Ethnic Categories**				
	Asian Indian	0.73	(0.61, 0.89)	0.001
	Chinese	0.63	(0.53, 0.75)	<0.001
	*NHW (Ref)*			

Based on 132,666 adult (age ≥ 18 years) NHIS participants from pooled cross-sectional data for years from 2012 through 2017, belonging to the racial/ethnic groups (Asian Indian, Chinese, and NHW) and did not have missing data on multimorbidity.
